# A new look at classical inequalities involving Banach lattice norms

**DOI:** 10.1186/s13660-017-1576-8

**Published:** 2017-12-08

**Authors:** Ludmila Nikolova, Lars-Erik Persson, Sanja Varošanec

**Affiliations:** 1 Department of Mathematics and Informatics, Sofia University, Sofia, Bulgaria; 2 Department of Engineering Sciences and Mathematics, Luleȧ University of Technology, Luleȧ, Sweden; 3 UiT, The Artic University of Norway, Tromsö, Norway; 4 RUDN University, Moscow, Russia; 5 Department of Mathematics, University of Zagreb, Zagreb, Croatia

**Keywords:** 26D10, 26D15, 46B70, 46E15, inequalities, continuous forms, Hölder’s inequality, Minkowski’s inequality, Popoviciu’s inequality, Bellman’s inequality, Beckenbach-Dresher’s inequality, Milne’s inequality, Banach function space, interpolation of families of spaces

## Abstract

Some classical inequalities are known also in a more general form of Banach lattice norms and/or in continuous forms (i.e., for ‘continuous’ many functions are involved instead of finite many as in the classical situation). The main aim of this paper is to initiate a more consequent study of classical inequalities in this more general frame. We already here contribute by discussing some results of this type and also by deriving some new results related to classical Popoviciu’s, Bellman’s and Beckenbach-Dresher’s inequalities.

## Introduction

Let $(Y, \Sigma, \nu)$ be a *σ*-finite measure space, and let $L^{0}(Y)$ denote the space of *ν*-measurable functions defined and being finite a.e. on *Y*. A Banach subspace $(E, \Vert \cdot \Vert )$ of $L^{0}(Y)$ is a Banach lattice (Banach function space) on $(Y, \Sigma, \nu)$ if, for every $x\in E$, $y \in L^{0}(Y)$, $\vert y \vert \leq \vert x \vert $, *ν*-a.e., it follows that $y\in E$ and $\Vert y \Vert \leq \Vert x \Vert $.

Moreover, the ‘convexification’ of *E*, denoted by $E^{p}$, $-\infty < p<\infty$, $p\neq0$ consists of all $x\in L^{0}(Y)$ satisfying

$$\Vert x \Vert _{E^{p}}:= \bigl( \bigl\Vert \vert x \vert ^{p} \bigr\Vert _{E} \bigr)^{\frac{1}{p}} < \infty. $$

For the case $p<0$, we assume that $x=x(t)\neq0$ for all $t\in Y$.

Some classical inequalities are known to hold also in the frame of such Banach lattice norms. See, for example, [1] and [2].

It is also known that some classical inequalities for finite many functions (like those of Hölder and Minkowski) can be generalized to hold for continuous (infinitely) many functions. For such results in $L_{p}$ and $l_{p}$ -spaces, we refer the reader to the recent article [3] and the references therein. We proved there the continuous versions of Popoviciu’s and Bellman’s inequalities.

However, there exists a generalization of Hölder’s inequality in both of these directions simultaneously, see [4] and also Lemma 2.2.

The main aim of this paper is to initiate a more consequent study of classical inequalities in this more general frame. Some known results of this type which we need in this paper can be found in our Section 2. We now shortly discuss some elementary forms of the inequalities we consider to generalize as new contributions in this paper.

We assume that $a_{i}$, $b_{i}$, $i=1,2,\ldots,n$, are positive numbers, $c_{1}$, $c_{2}$ are positive numbers and *f* and *g* are positive functions on $(Y, \Sigma, \nu)$.

*A. Popoviciu’s inequality*: Let $p,q \geq1$, $\frac{1}{p}+\frac {1}{q}=1$. In the most elementary form it reads (see [5]): If $c_{1}- (\sum_{i=1}^{n}a_{i}^{p} )^{\frac{1}{p}}>0$ and $c_{2}- (\sum_{i=1}^{n}b_{i}^{q} )^{\frac{1}{q}}>0$, then

1.1 $$ \Biggl(c_{1}^{p}-\sum _{i=1}^{n}a_{i}^{p} \Biggr)^{\frac{1}{p}} \Biggl(c_{2}^{q}-\sum _{i=1}^{n}b_{i}^{q} \Biggr)^{\frac{1}{q}}\leq c_{1}c_{2} -\sum _{i=1}^{n}a_{i}b_{i}. $$

A generalization of this inequality reads: If $c_{1}-(\int_{Y}f^{p}(y) \,d\nu (y))^{\frac{1}{p}}>0$ and $c_{2}- [4](\int_{Y}g^{q}(y) \,d\nu(y))^{\frac {1}{q}}>0$, then

1.2 $$ \begin{aligned}[b] & \biggl(c_{1}^{p}- \int_{Y}f^{p}(y) \,d\nu(y) \biggr)^{\frac{1}{p}} \biggl(c_{2}^{q}- \int _{Y}g^{q}(y) \,d\nu(y) \biggr)^{\frac{1}{q}} \\ &\quad \leq c_{1}c_{2}- \int_{Y}f(y)g(y) \,d\nu(y). \end{aligned} $$

For more general forms, see, e.g., Theorem 3.3 which in particular shows that ‘>’ in the assumptions of (1.1) and (1.2) can be replaced by ‘≥’. Some continuous forms of (1.1) and (1.2) were recently proved in [3].

In Section 3 of this paper we present, prove and apply our main results concerning Popoviciu’s inequality (see Theorems 3.1 and 3.3).

*B. Bellman’s inequality*: The original form of Bellman’s inequality reads (see [6] and also [7]): If $p\geq1$ and $c_{1}- (\sum_{i=1}^{n}a_{i}^{p} )^{\frac{1}{p}}>0$ and $c_{2}- (\sum_{i=1}^{n}b_{i}^{p} )^{\frac{1}{p}}>0$, then

1.3 $$ \Biggl(c_{1}^{p}-\sum _{i=1}^{n}a_{i}^{p} \Biggr)^{\frac{1}{p}}+ \Biggl(c_{2}^{p}-\sum _{i=1}^{n}b_{i}^{p} \Biggr)^{\frac{1}{p}}\leq \Biggl((c_{1}+c_{2})^{p} -\sum_{i=1}^{n}(a_{i}+b_{i})^{p} \Biggr)^{\frac{1}{p}}. $$

There is also a more general integral form of this inequality, namely: If $c_{1}- [4] (\int_{Y}f^{p}(y) \,d\nu(y) )^{\frac{1}{p}}>0$ and $c_{2}- (\int_{Y}g^{p}(y) \,d\nu(y) )^{\frac{1}{p}}>0$, then

1.4 $$ \begin{aligned}[b] &\biggl(c_{1}^{p}- \int_{Y}f^{p}(y) \,d\nu(y) \biggr)^{\frac{1}{p}}+ \biggl(c_{2}^{p}- \int _{Y}g^{p}(y) \,d\nu(y) \biggr)^{\frac{1}{p}} \\ &\quad \leq \biggl((c_{1}+c_{2})^{p} - \int _{Y}\bigl(f(y)+g(y)\bigr)^{p} \,d\nu(y) \biggr)^{\frac{1}{p}}, \end{aligned} $$

which holds under proper conditions. For more general forms, see, e.g., Theorem 4.3. Also here our result shows in particular that ‘>’ in the assumptions of (1.3) and (1.4) can be replaced by ‘≥’. Some continuous forms of (1.3) and (1.4) were recently proved in [3].

Our main results related to this inequality are proved and discussed in Section 4. We remark that obviously Popoviciu’s and Bellman’s inequalities may be regarded as a type of reversed inequalities of Hölder’s and Minkowski’s inequalities, respectively.

*C. Beckenbach-Dresher’s inequality*: In its most elementary form it reads: If $p\geq1$, $\beta\leq1 \leq\alpha$; $\beta\neq0$, then

1.5 $$ \frac{ (\sum_{i=1}^{n}(a_{i}+b_{i})^{\alpha})^{p/\alpha}}{ (\sum_{i=1}^{n}(a_{i}+b_{i})^{\beta})^{(p-1)/\beta}} \leq\frac{ (\sum_{i=1}^{n}a_{i}^{\alpha})^{p/\alpha}}{ (\sum_{i=1}^{n}a_{i}^{\beta})^{(p-1)/\beta}}+ \frac{ (\sum_{i=1}^{n}b_{i}^{\alpha})^{p/\alpha}}{ (\sum_{i=1}^{n}b_{i}^{\beta})^{(p-1)/\beta}}. $$

Especially for the case $p=\alpha/(\alpha-\beta)$, $\alpha\neq\beta$, we obtain the triangle inequality for the so-called Gini-means *G* defined by

$$G=G(\alpha,\beta)= \biggl(\frac{\sum_{i=1}^{n} a_{i}^{\alpha}}{\sum_{i=1}^{n}a_{i}^{\beta}} \biggr)^{1/(\alpha-\beta)},\quad \alpha\neq \beta. $$

There are many generalizations of inequality (1.5). Of special importance as the background for this paper, we mention [2], where also a version for the spaces $E^{p}$ is included.

In Section 5 of the present paper, we derive a new version of (1.5) which is both ‘continuous’ (containing infinitely many functions, e.g., sequences) and involving Banach lattice norms (see Theorem 5.1). Moreover, we also derive a type of reversed inequality of the same general form (see Theorem 5.4). Finally, Section 6 is reserved for some concluding remarks and results. Especially, we present new Popoviciu’s inequality in the case of infinite interpolation families (see Theorem 6.1), and the connection to Milne’s inequality is pointed out (see Section 6.2).

## Preliminaries

It is known that if $\Vert \cdot \Vert _{E}$ is a Banach function norm, then $\Vert f(x,\cdot) \Vert _{E}$ need not be a measurable function. But it is also known that if *E* has the Fatou property, then indeed $\Vert f(x,\cdot) \Vert _{E}$ is measurable (see [8]). Therefore, for simplicity, we assume that the considered Banach function spaces have the Fatou property. It is also known that in this situation *E* is a perfect space, i.e., $E=E''$, where $E''$ denotes the second associate space of *E*.

We need the following simple generalization of Hölder’s inequality.

### Lemma 2.1

*Let* $p,q \neq0$, $\frac{1}{p}+\frac{1}{q}=1$. *If* $p>1$, *then*

2.1 $$ \Vert fg \Vert _{E}\leq \Vert f \Vert _{E^{p}} \Vert g \Vert _{E^{q}}. $$

*If* $p<1$, *then* (2.1) *holds in the reverse direction*. *We have equality in* (2.1) *when* $g=cf^{p-1}$.

A simple proof of this lemma in an even general symmetric form can be found in [1], p. 369.

We also need the following more general form of Hölder’s inequality (both continuous and involving Banach function norms).

### Lemma 2.2

*Let* $E=E''$, $0< b\leq\infty$, $p(x)>0$, $u(x)\geq0$ *be measurable and define* *p* *by*

2.2 $$ \frac{1}{p}= \int_{0}^{b}\frac{u(x)}{p(x)} \,dx, $$

*where* $\int_{0}^{b}u(x) \,dx=1$, *then*

2.3 $$ \biggl\Vert \exp \biggl( \int_{0}^{b} \log \bigl(f(x,y)\bigr) u(x) \,dx \biggr) \biggr\Vert _{E^{p}}\leq\exp ( \int_{0}^{b} \log \Vert f\bigl(x,y \Vert _{E^{p(x)}} u(x) \,dx \bigr). $$

A proof of this result can be found in [4].

We note that (2.3) is an inequality between generalized geometric means. We also need the following inequality.

### Lemma 2.3

*Let* $f(x)$, $g(x)$, $u(x)$ *be positive and* $\int _{X}u(x) \,d\mu(x)=1$. *Then*

$$\begin{gathered} \exp \biggl[ \int_{X} \log\bigl(f(x)\bigr) u(x) \,d\mu(x) \biggr]+\exp \biggl[ \int_{X} \log \bigl(g(x)\bigr) u(x) \,d\mu(x) \biggr] \\ \quad \leq\exp \biggl[ \int_{X} \log \bigl(f(x)+g(x)\bigr) u(x) \,d\mu(x) \biggr]. \end{gathered} $$

See, e.g., [9]. Another proof can be done by just using reversed form of suitable generalizations of Beckenbach-Dresher’s inequality with $p=\alpha/ (\alpha-\beta) $ and letting $\alpha,\beta\rightarrow 0$ in the corresponding generalized Gini-means $G(\alpha,\beta)$.

We also need the following analogous version of Minkowski’s inequality.

### Lemma 2.4

*Let* $E=E''$, *let* $p\geq1$. *If* $f(x,y)\geq0$ *on* $X\times Y$, *then*

2.4 $$ \biggl\Vert \int_{X} f(x,y) \,dx \biggr\Vert _{E^{p}}\leq \int_{X} \bigl\Vert f(x,y) \bigr\Vert _{E^{p}} \,dx $$

*or*, *equivalently*,

2.5 $$ \biggl\Vert \biggl( \int_{X} f(x,y) \,dx \biggr)^{p} \biggr\Vert _{E}^{\frac{1}{p}}\leq \int_{X} \bigl\Vert f^{p}(x,y) \bigr\Vert _{E}^{\frac{1}{p}} \,dx. $$

Since E has the Fatou property and $p\geq1$, we have that $E^{p}$ has the Fatou property, i.e., it is a perfect space, and the proof can be found in [10], Chapter 2. Note that in the case $E=L_{1}(Y)$ this is just the classical integral Minkowski inequality.

Remember that the Banach lattice *E* is *p*-convex or *q*-concave if there exists a positive constant *M* such that, for every finite set $x_{1}, x_{2}, \ldots, x_{n}$ of elements in *E*, we have

$$\Biggl\Vert \Biggl(\sum_{i=1}^{n} \vert x_{i} \vert ^{p}\Biggr)^{1/p} \Biggr\Vert _{E}\leq M\Biggl(\sum_{i=1}^{n} \Vert x_{i} \Vert _{E}^{p} \Biggr)^{1/p}, $$

or

$$\Biggl(\sum_{i=1}^{n} \Vert x_{i} \Vert _{E}^{q}\Biggr)^{1/q}\leq M \Biggl\Vert \Biggl(\sum_{i=1}^{n} \vert x_{i} \vert ^{q}\Biggr)^{1/q} \Biggr\Vert _{E}, $$

respectively.

The smallest *M* satisfying the corresponding inequality is called constant of *p*-convexity, respectively, of *q*-concavity.

In [11] the following fact appears: Let *ρ* and *λ* be function norms with the Fatou property and assume that there exists $1\leq p \leq\infty$ such that *ρ* is p-convex and *λ* is p-concave. Then there exists a constant C such that, for all measurable $f(x, y)$, we have

2.6 $$ \rho(\lambda\bigl(f(x,\cdot)\bigr) \leq C\lambda(\rho\bigl(f( \cdot,y)\bigr). $$

If we take $\rho(h)$ to be $\int_{X} h(x) \,dx$ which is 1-convex with constant of convexity equal to 1 and $\lambda= \Vert \, \Vert _{E}$, where *E* is 1-concave with constant of concavity equal to *M*, and follow the part of the proof of (2.6) in which the constant of convexity of the norm $\rho(\cdot)$ is equal to 1, we find that

2.7 $$ M \biggl\Vert \int_{X}f(x,\cdot) \,dx \biggr\Vert _{E} \geq \int_{X} \bigl\Vert f(x,\cdot) \bigr\Vert _{E} \,dx. $$

### Lemma 2.5

*If* *E* *has the Fatou property and is* 1-*concave with constant of concavity equal to* *M*, $p<1 $, $p\neq0$, *then*

2.8 $$ M \biggl\Vert \int_{X} f(x,y) \,dx \biggr\Vert _{E^{p}}\geq \int_{X} \bigl\Vert f(x,y) \bigr\Vert _{E^{p}} \,dx. $$

### Proof

We follow the idea in the proof of Theorem 4.1.b) in [1] by using Lemma 2.1. In details, using Theorem 3.1 b) from [1] and (2.7), we have

$$\begin{aligned}& M \biggl\Vert \int_{X} f(x,y) \,dx \biggr\Vert _{E^{p}}\\ &\quad = \inf _{ \Vert z \Vert _{E^{q}}=1} M \biggl\Vert \int_{X} f(x,y)z \,dx \biggr\Vert _{E} \\ &\quad \geq \inf_{ \Vert z \Vert _{E^{q}}=1} \int_{X} \bigl\Vert f(x,y)z \bigr\Vert _{E} \,dx\geq \int_{X}\inf_{ \Vert z \Vert _{E^{q}}=1} \bigl\Vert f(x,y)z \bigr\Vert _{E} \,dx= \int_{X} \bigl\Vert f(x,y) \bigr\Vert _{E^{p}} \,dx. \end{aligned} $$

Here, $q=p/(p-1)$, $z=z(y) \in E^{q}$, $z(y)> 0$. □

## Popoviciu type inequalities involving Banach function norms

Our first main result reads as follows.

### Theorem 3.1

*Let* *E* *be a Banach function space such that* $E''=E $ *and* $0< b\leq\infty$. *Let* *p* *be defined by* (2.2) *and* $\int _{0}^{b}u(x) \,dx=1$. *If* $f(x,y)$ *and* $p(x)$ *are positive and* $f_{0}(x)> \Vert f(x,y) \Vert _{E^{p(x)}} >0$, *then*

3.1 $$ \begin{gathered}[b] \exp \biggl( \int_{0}^{b} \log\bigl(f_{0}(x)\bigr) u(x) \,dx \biggr)- \biggl\Vert \exp \biggl( \int_{0}^{b} \log\bigl(f(x,y)\bigr)u(x) \,dx \biggr) \biggr\Vert _{E^{p}} \\ \quad {}-\exp \biggl[ \int_{0}^{b} \log \bigl(f_{0}(x)- \bigl\Vert f(x,y) \bigr\Vert _{E^{p(x)}} \bigr)u(x) \,dx \biggr]\geq0, \end{gathered} $$

*provided that all integrals which occur in* (3.1) *exist*.

### Proof

We use Lemma 2.3 with $X=[0,b)$, $f(x)=f_{0}(x)- \Vert f(x,\cdot) \Vert _{E^{p(x)}}$, $g(x)= \Vert f(x,\cdot) \Vert _{E^{p(x)}}$ and find that

$$\begin{aligned} \exp \biggl( \int_{0}^{b} \log\bigl(f_{0}(x)\bigr) u(x) \,dx \biggr)&\geq\exp \biggl( \int _{0}^{b} \log\bigl( \bigl\Vert f(x,\cdot) \bigr\Vert _{E^{p(x)}}\bigr)u(x) \,dx \biggr) \\ &\quad {}+\exp \biggl[ \int_{0}^{b} \log \bigl(f_{0}(x)- \bigl\Vert f(x,\cdot) \bigr\Vert _{E^{p(x)}} \bigr)u(x) \,dx \biggr]. \end{aligned} $$

Next we use Lemma 2.2 to conclude that

$$\exp \biggl( \int_{0}^{b} \log\bigl( \bigl\Vert f(x,\cdot) \bigr\Vert _{E^{p(x)}}\bigr)u(x) \,dx \biggr)\geq \biggl\Vert \exp \biggl( \int_{a}^{b} \log\bigl(f(x,y)\bigr)u(x) \,dx \biggr) \biggr\Vert _{E^{p}}. $$

We combine the above two inequalities and obtain (3.1). The proof is complete. □

### Example 3.2

(a) Applying Theorem 3.1 with $E=L_{1}(Y,v )$, we find that

$$\begin{gathered} \exp \biggl( \int_{0}^{b} \log\bigl(f_{0}(x)\bigr) u(x) \,dx \biggr) \\ \qquad {}- \biggl\{ \int_{Y} \biggl[\exp \biggl( \int_{0}^{b} \log\bigl(f(x,y)\bigr)u(x) \,dx \biggr) \biggr]^{p} v(y) \,dy \biggr\} ^{\frac{1}{p}} \\ \quad \geq\exp \biggl[ \int_{0}^{b} \log\biggl(f_{0}(x)- \biggl( \int_{Y} f^{p(x)}(x,y)v(y) \,dy \biggr)^{\frac{1}{p(x)}} \biggr)u(x) \,dx \biggr], \end{gathered} $$

which in the case $p(x)\equiv1$ was proved in [3].

(b) If $p(x)\equiv1$, then (3.1) reads

3.2 $$ \begin{aligned}[b] &\exp \biggl( \int_{0}^{b} \log \bigl(f_{0}(x) \bigr)u(x) \,dx \biggr)- \biggl\Vert \exp \biggl( \int_{0}^{b} \log\bigl(f(x,y) \bigr)u(x) \,dx \biggr) \biggr\Vert _{E} \\ &\quad {} -\exp \biggl[ \int_{0}^{b} \log \bigl(f_{0}(x)- \bigl\Vert f(x,y) \bigr\Vert _{E} \bigr)u(x) \,dx \biggr]\geq0, \end{aligned} $$

which in the case $E=L_{1}(Y)$ was proved in [3].

Next we state the following complementary result.

### Theorem 3.3

*Let* *E* *be a Banach function space*, *let* $f,g\geq0$ *and* $p,q\neq0$, *where* $\frac{1}{p}+\frac{1}{q}=1$.

- *Let* $p\geq1$. *If* $c_{1}^{p}- \Vert f^{p} \Vert _{E}\geq0$, $c_{2}^{q}- \Vert g^{q} \Vert _{E}\geq0$, *then*
  3.3 $$ c_{1}c_{2}- \Vert fg \Vert _{E} - \bigl(c_{1}^{p}- \bigl\Vert f^{p} \bigr\Vert _{E}\bigr)^{1/p} \bigl(c_{2}^{q}- \bigl\Vert g^{q} \bigr\Vert _{E}\bigr)^{1/q}\geq0. $$
- *Let* $0< p<1$. *If* $\Vert g^{q} \Vert _{E}>0$, $c_{2}^{q}- \Vert g^{q} \Vert _{E}>0$, *then reverse inequality* (3.3) *holds*.
- *Let* $p<0$. *If* $\Vert f^{p} \Vert >0$, $c_{1}^{p}- \Vert f^{p} \Vert _{E} >0$, *then reverse inequality* (3.3) *holds*.

### Proof

(a) Let $p,q>0$. Let $c_{1}^{p}- \Vert f^{p} \Vert _{E}$, $g_{0}^{q}- \Vert g^{q} \Vert _{E}$ be strictly positive. Let $X_{1} \cup X_{2}=[0,b)$, let $X_{1} \cap X_{2}$ be empty, $\int_{X_{1}}u(x) \,dx =\frac {1}{p}$, $\int_{X_{2}}u(x) \,dx =\frac{1}{q}$. We can get the result like a corollary from inequality (3.2), by taking $f_{0}(x)=c_{1}^{p}$ for $x\in X_{1}$ and $f_{0}(x)=c_{2}^{q}$ and $f(x,y)=f^{p}(y)$ for $x\in X_{1}$, $f(x,y)=g^{q}(y)$ for $x\in X_{2}$.

If, for instance, $c_{1}^{p}- \Vert f^{p} \Vert _{E}=0$, $g_{0}^{q}- \Vert g^{q} \Vert _{E}\geq0$, we have to show that $c_{1}c_{2}\geq \Vert fg \Vert $. For this purpose we just use Lemma 2.1, namely

$$\Vert fg \Vert _{E}\leq \bigl\Vert f^{p} \bigr\Vert _{E}^{\frac{1}{p}} \bigl\Vert g^{q} \bigr\Vert _{E}^{\frac{1}{q}}\leq c_{1}c_{2}. $$

(b) The case $0< p<1$ can be treated by using the same Lemma 2.1, which says that

$$\Vert fg \Vert _{E}\geq \bigl\Vert f^{p} \bigr\Vert _{E}^{\frac{1}{p}} \bigl\Vert g^{q} \bigr\Vert _{E}^{\frac{1}{q}} $$

in this case. If we put

$$x=\bigl(c_{1}^{p}- \bigl\Vert f^{p} \bigr\Vert _{E}\bigr)^{1/p}, y=\bigl( \bigl\Vert f^{p} \bigr\Vert _{E}\bigr)^{1/p}, z= \bigl(g_{0}^{q}- \bigl\Vert g^{q}\bigr) \bigr\Vert _{E}^{1/q}, t=\bigl( \bigl\Vert g^{q} \bigr\Vert _{E} \bigr)^{1/q}, $$

we have

$$yt\leq \Vert fg \Vert _{E}, $$

which together with Hölder’s inequality

$$xz+yt\geq\bigl(x^{p}+y^{p}\bigr)^{\frac{1}{p}} \bigl(z^{q}+t^{q}\bigr)^{\frac{1}{q}} $$

gives us the wanted inequality.

(c) The case $p<0$ can be proved similarly (just interchange the roles of *f* and *g* and *p* and *q*, respectively). □

### Remark 3.4

Note that Theorem 3.3 in particular means that inequalities (1.1) and (1.2) hold also if ‘>’ in these inequalities are replaced by ‘≥’.

We also state a generalization of Theorem 3.3(a).

### Corollary 3.5

*Let* *E* *be a Banach function space*, *let* *f*, *g*, $c_{1}^{p}- \Vert f^{p} \Vert _{E^{r}}\geq0$, $c_{2}^{q}- \Vert g^{q} \Vert _{E^{s}}\geq0$, *where* $p,q,r,s>0 $, $\frac{1}{pr}+\frac{1}{qs}=1$. *Then*

3.4 $$ c_{1}c_{2}- \Vert fg \Vert _{E} - \bigl(c_{1}^{p}- \bigl\Vert f^{p} \bigr\Vert _{E^{r}}\bigr)^{1/p} \bigl(c_{2}^{q}- \bigl\Vert g^{q} \bigr\Vert _{E^{s}}\bigr)^{1/q}\geq0. $$

### Proof

In the case when $c_{1}^{p}- \Vert f^{p} \Vert _{E}$, $g_{0}^{q}- \Vert g^{q} \Vert _{E}$ are strictly positive, we can get like corollary from Theorem 3.1. First we take $u(x)=1$, $d\mu(x)= dx$ and, if $\int_{0}^{b}\frac{1}{p(x)} \,dx=1$, we get

3.5 $$ \begin{gathered}[b] \exp \biggl( \int_{0}^{b} \log\bigl(f_{0}(x)\bigr) \,dx \biggr)- \biggl\Vert \exp \biggl( \int_{0}^{b} \log\bigl(f(x,y) \bigr) \,dx \biggr) \biggr\Vert _{E} \\ \quad {}-\exp \biggl[ \int_{0}^{b} \log \bigl(f_{0}(x)- \bigl\Vert f(x,y) \bigr\Vert _{E^{p(x)}} \bigr) \,dx \biggr]\geq0. \end{gathered} $$

Then we take $f_{0}(x)=c_{1}^{p}$ for $x\in X_{1}$, $f_{0}(x)=c_{2}^{q}$ for $x\in X_{2}$, $f(x,y)=f^{p}(y)$ for $x\in X_{1}$, $f(x,y)=g^{q}(y)$ for $x\in X_{2}$, where $X_{1} \cup X_{2}=[0,b)$, $X_{1} \cap X_{2}$ is empty, $\int _{X_{1}}u(x) \,dx =\frac{1}{p}$, $\int_{X_{2}}u(x) \,dx =\frac{1}{q}$, $p(x)=r $ for $x\in X_{1}$ and $p(x)=s $ for $x\in X_{2} $.

Look, for instance, to the third expression in inequality (3.5)

$$\begin{gathered} \exp \biggl[ \int_{a}^{b} \log \bigl(f_{0}(x)- \bigl\Vert f(x,y) \bigr\Vert _{E^{p(x)}} \bigr)u(x) \,dx \biggr] \\ \quad =\exp \biggl[ \int_{X_{1}} \log \bigl(c_{1}^{p}- \bigl\Vert f^{p}(y) \bigr\Vert _{E^{r}} \bigr)u(x) \,dx+ \int_{X_{2}} \log \bigl(c_{2}^{q}- \bigl\Vert f^{q}(y) \bigr\Vert _{E^{s}} \bigr)u(x) \,dx \biggr] \\ \quad = \exp \biggl[ \log \bigl(c_{1}^{p}- \bigl\Vert f^{p}(\cdot) \bigr\Vert _{E^{r}} \bigr)\frac{1}{p}+\log \bigl(c_{2}^{q}- \bigl\Vert g^{q}(\cdot) \bigr\Vert _{E^{s}} \bigr)\frac{1}{q} \biggr] \\ \quad =\bigl(c_{1}^{p}- \bigl\Vert f^{p} \bigr\Vert _{E^{r}}\bigr)^{1/p} \bigl(c_{2}^{q}- \bigl\Vert g^{q} \bigr\Vert _{E^{s}}\bigr)^{1/q}, \end{gathered} $$

which is the third expression in (3.4). Even simpler we see that the two first terms in (3.5) coincide with the corresponding terms in (3.4).

If, for instance, $c_{1}^{p}- \Vert f^{p} \Vert _{E^{r}}=0$, $c_{2}^{q}- \Vert g^{q} \Vert _{E^{s}}\geq0$, we have to show that $c_{1}c_{2}\geq \Vert fg \Vert $. For the purpose, we just use Lemma 2.1, namely

$$\Vert fg \Vert _{E}\leq \bigl\Vert f^{p} \bigr\Vert _{E^{r}}^{\frac{1}{p}} \bigl\Vert g^{q} \bigr\Vert _{E^{s}}^{\frac{1}{q}}\leq c_{1}c_{2}. $$

The proof is complete. □

### Remark 3.6

1. Since

3.6 $$ (a-b)^{r}\geq a^{r}-b^{r} \quad \mbox{when } a>b>0, 0< r< 1, $$

by putting $a=c_{1}^{p}$, $b= \Vert f^{pr} \Vert _{E}^{1/r}$, we get

$$\bigl(c_{1}^{p} - \bigl\Vert f^{pr} \bigr\Vert _{E}^{1/r} \bigr)^{r} \geq c_{1}^{pr} - \bigl\Vert f^{pr} \bigr\Vert _{E}. $$

Hence, for $0< r,s <1$, the following chain of inequalities holds:

$$\begin{aligned} c_{1}c_{2} - \Vert fg \Vert _{E} &\geq\bigl(c_{1}^{p} - \bigl\Vert f^{p} \bigr\Vert _{E^{r}} \bigr)^{1/p} \bigl(c_{2}^{q} - \bigl\Vert g^{q} \bigr\Vert _{E^{s}} \bigr)^{1/q} \\ & \geq \bigl(c_{1}^{pr} - \bigl\Vert f^{pr} \bigr\Vert _{E} \bigr)^{1/pr} \bigl(c_{2}^{qs} - \bigl\Vert g^{qs} \bigr\Vert _{E} \bigr)^{1/qs}. \end{aligned} $$

If we compare inequalities (3.4) and (3.3), we can see that in the case $0< r,s<1$ inequality (3.4) is better than inequality (3.3). Moreover, since in the case $r,s>1$ inequality (3.6) holds in the reversed direction when $r>1$, in the case $r,s>1$ inequality (3.3) is stronger than inequality (3.4).

2. Note that here we do not need the condition $E''=E$ because of Remark after Theorem 4.1 from [4].

## Bellman type inequalities involving Banach function norms

Our first main result in this case reads as follows.

### Theorem 4.1

*Let* *X* *and* *Y* *be measure spaces*, *let* $f(x,y)$ *be a positive measurable function on* $X\times Y$ *and assume that* $p\geq1$ *and* $f_{0}(x)$ *is a function on* *X* *such that* $f_{0}^{p}(x)> \Vert f^{p}(x,\cdot) \Vert _{E}$, *where* *E* *is a Banach function space on* *Y* *for all* $x\in X$. *Assume that* *E* *has the Fatou property*. *Then*

4.1 $$ \begin{aligned}[b] & \biggl( \int_{X} \bigl[f^{p}_{0}(x)- \bigl\Vert f^{p}(x,\cdot) \bigr\Vert _{E} \bigr]^{\frac{1}{p}} \,dx \biggr)^{p} \\ &\quad \leq \biggl[ \int_{X}f_{0}(x) \,dx \biggr]^{p}- \biggl\Vert \biggl[ \int_{X} f(x,\cdot) \,dx \biggr]^{p} \biggr\Vert _{E}, \end{aligned} $$

*provided that all integrals exist*.

*If* *E* *is* 1-*concave with constant of concavity* 1, $0< p<1$ *or* $p<0$ *and* $\Vert f^{p}(x,\cdot) \Vert _{E}>0$, *then inequality* (4.1) *holds in the reverse direction*.

### Proof

Let $p\geq1$. We consider the following form of Minkowski’s integral inequality:

4.2 $$ \begin{aligned}[b] & \biggl( \int_{Y} \biggl( \int_{X} f(x,y)u(x) \,d\mu(x) \biggr)^{p}v(y) \,d \nu(y) \biggr)^{\frac{1}{p}} \\ &\quad \leq \int_{X} \biggl( \int_{Y} f^{p}(x,y) v(y) \,d\nu(y) \biggr)^{\frac{1}{p}} u(x) \,d\mu(x) \end{aligned} $$

for the special case when $Y=Y_{1}\cup Y_{2}$, $Y_{1}\cap Y_{2}$ is empty, $f(x,y)=a(x)$ on $Y_{1}$, $f(x,y)=b(x)$ on $Y_{2}$ and $\int_{Y_{1}}v(y) \,d \nu(y) =\int_{Y_{2}}v(y) \,d \nu(y)=1 $, $u(x) \,d\mu (x)= dx $ and get

$$\biggl( \int_{X} a(x) \,dx \biggr)^{p} + \biggl( \int_{X} b(x) \,dx \biggr)^{p}\leq \biggl[ \int_{X} \bigl(a^{p}(x)+b^{p}(x) \bigr)^{\frac{1}{p}} \,dx \biggr]^{p}. $$

We choose

$$a(x)= \bigl[f^{p}_{0}(x)- \bigl\Vert f^{p}(x, \cdot) \bigr\Vert _{E} \bigr]^{\frac {1}{p}},\qquad b(x)= \bigl[ \bigl\Vert f^{p}(x,\cdot) \bigr\Vert _{E} \bigr]^{\frac{1}{p}} $$

and obtain that

$$\begin{gathered} \biggl( \int_{X} \bigl[f^{p}_{0}(x)- \bigl\Vert f^{p}(x,\cdot) \bigr\Vert _{E} \bigr]^{\frac{1}{p}} \,dx \biggr)^{p} \\ \quad {}+ \biggl( \int_{X} \bigl\Vert f^{p}(x,\cdot) \bigr\Vert _{E} ^{\frac{1}{p}} \,dx \biggr)^{p}\leq \biggl[ \int_{X} f_{0}(x) \,dx \biggr]^{p}:= I_{1}. \end{gathered} $$

Next, by using (2.5) to the second term in the above inequality, we find that

4.3 $$ \begin{aligned}[b] I_{1}&\geq \biggl\Vert \biggl( \int_{X} f(x,\cdot) \,dx \biggr)^{p} \biggr\Vert _{E} \\ &\quad {}+ \biggl( \int_{X} \bigl[f^{p}_{0}(x)- \bigl\Vert f^{p}(x,\cdot) \bigr\Vert _{E} \bigr]^{\frac{1}{p}} \,dx \biggr)^{p}. \end{aligned} $$

If $0< p<1$, then first we use reverse to inequality (4.2) and then instead of (2.5) we use (2.8) for $M=1$.

The proof in the case $p<0$ is similar. □

### Remark 4.2

Inequality (4.1) can be written as follows:

$$\begin{gathered} \biggl( \int_{X} \bigl[f^{p}_{0}(x)- \bigl\Vert f(x,\cdot) \bigr\Vert ^{p}_{E^{p}} \bigr]^{\frac{1}{p}} \,dx \biggr)^{p} \\ \quad \leq \biggl[ \int_{X}f_{0}(x) \,dx \biggr]^{p}- \biggl\Vert \int_{X} f(x,\cdot) \,dx \biggr\Vert ^{p}_{E^{p}}. \end{gathered} $$

If $E=L_{1}$, then we get the result of the first part of the continuous Bellman inequality for $p\geq1$ proved in [3], Theorem 3.1.

Next, we state the following Bellman type inequalities.

### Theorem 4.3

- *Let* *E* *be a Banach function space*, *let* $f,g>0$, $p\geq1$, $c_{1}^{p}- \Vert f^{p} \Vert _{E}\geq0$, $c_{2}^{p}- \Vert g^{p} \Vert _{E}\geq0$. *Then*
  4.4 $$ \bigl(\bigl(c_{1}^{p}- \bigl\Vert f^{p} \bigr\Vert _{E}\bigr)^{1/p} + \bigl(c_{2}^{p}- \bigl\Vert g^{p} \bigr\Vert _{E}\bigr)^{1/p}\bigr)^{p} \leq (c_{1}+c_{2})^{p}- \bigl\Vert (f+g)^{p} \bigr\Vert _{E}. $$
- *If* *E* *is an arbitrary* 1-*concave lattice with constant of concavity* 1, $0< p<1$ *or* $p<0$ *and* $c_{1},f>0$, $c_{1}^{p}- \Vert f^{p} \Vert _{E}>0$, $c_{2}>0$, $g>0$, $c_{2}^{p}- \Vert g^{p} \Vert _{E}>0$, *then reverse inequality* (4.4) *holds*.

### Proof

(a) In view of Theorem 4.1, from [1] we have the following variant of Minkowski’s inequality: If $p\geq1$, then

4.5 $$ \Biggl\Vert \sum_{1}^{n}x_{i} \Biggr\Vert _{E^{p}}\leq\sum_{1}^{n} \Vert x_{i} \Vert _{E^{p}}. $$

We follow the idea of the proof of Theorem 4.29 from [12] using the discrete Minkowski inequality

4.6 $$ \bigl((a_{1}+b_{1})^{p}+ (a_{2}+b_{2})^{p}\bigr)^{\frac{1}{p}}\leq \bigl(a_{1}^{p}+ a_{2}^{p} \bigr)^{\frac{1}{p}} +\bigl(b_{1}^{p}+ b_{2}^{p}\bigr)^{\frac{1}{p}} $$

with

$$a_{1}=\bigl(c_{1}^{p}- \bigl\Vert f^{p} \bigr\Vert _{E}\bigr)^{1/p}, b_{1}=\bigl(c_{2}^{p}- \bigl\Vert g^{p} \bigr\Vert _{E}\bigr)^{1/p}, a_{2}=\bigl( \bigl\Vert f^{p} \bigr\Vert _{E} \bigr)^{1/p}, b_{2}=\bigl( \bigl\Vert g^{p} \bigr\Vert _{E}\bigr)^{1/p}. $$

We note that the right-hand side in (4.6) is equal to $c_{1}+c_{2}$, $(a_{1}+b_{1})^{p}$ coincides with the term on the left-hand side in (4.4) and, by (4.5),

$$a_{2}+b_{2}= \Vert f \Vert _{E^{p}}+ \Vert g \Vert _{E^{p}}\geq \Vert f+g \Vert _{E^{p}}= \bigl\Vert (f+g)^{p} \bigr\Vert _{E}^{\frac{1}{p}}. $$

By using these facts and taking *p*th power of both sides in (4.6), we get (4.4).

(b) All inequalities above hold in the reverse direction in this case, and the proof follows by just doing obvious modifications of the proof of (a). □

### Remark 4.4

Note that similarly as in our previous section, Theorem 4.1 in particular means that inequalities (1.3) and (1.4) hold also if ‘>’ in the statements of these inequalities are replaced by ‘≥’.

## Direct and reverse Beckenbach-Dresher type inequalities involving Banach function norms

The following result concerning Beckenbach-Dresher’s inequality was announced in [13]. For completeness, we give here also the proof.

### Theorem 5.1

*Let E*, *F be Banach function spaces with the Fatou property*. *If* $0< u<1$, $0< p,q\leq1$ *and* *E* *is* 1-*concave with constant of concavity equal to* *M*, *F* *is* 1-*concave with constant of concavity equal to* *N*, *then the inequality*

5.1 $$ \frac{ \Vert \int_{X} f(x,\cdot) \,dx \Vert _{E^{p}}^{u}}{ \Vert \int_{X} g(x,\cdot) \,dx \Vert _{F^{q}}^{u-1}}\geq C \int_{X}\frac{ \Vert f(x,\cdot) \Vert _{E^{p}}^{u}}{ \Vert g(x,\cdot) \Vert _{F^{q}} ^{u-1}} \,dx $$

*holds with* $C=M^{-u}N^{u-1}$, *providing all above integrals exist*.

*If* $u>1$, $q\leq1\leq p$, $q\neq0$ *and* *F* *is* 1-*concave with constant of concavity equal to* *N*, *then inequality* (5.1) *holds in the reverse direction with* $C=N^{u-1}$.

*If* $u<0$, $p \leq1\leq q$, $p\neq0$ *and* *E* *is* 1-*concave with constant of concavity equal to* *M*, *then inequality* (5.1) *holds in the reverse direction with* $C=M^{-u}$.

### Proof

In the proof we will use (2.5), (2.8) and Hölder’s or reverse Hölder’s inequalities.

Let $0< u<1$, *E* be 1-concave with constant of concavity equal to *M*, and let *F* be 1-concave with constant of concavity equal to *N*. Then

$$\begin{aligned} \frac{ \Vert \int_{X} f(x,\cdot) \,dx \Vert _{E^{p}}^{u}}{ \Vert \int_{X} g(x,\cdot) \,dx \Vert _{F^{q}}^{u-1}}&\geq \frac{M^{-u} (\int_{X} \Vert f(x,\cdot) \Vert _{E^{p}} \,dx )^{u}}{N^{1-u} (\int_{X} \Vert g(x,\cdot) \Vert _{F^{q}} \,dx )^{u-1}} \\ &\geq M^{-u}N^{u-1} \int_{X}\frac{ \Vert f(x,\cdot) \Vert _{E^{p}}^{u}}{ \Vert g(x,\cdot) \Vert _{F^{q}}^{u-1}} \,dx. \end{aligned} $$

Let $u>1$, *E* be just a Banach function space, and let *F* be 1-concave with constant of concavity equal to *N*. Then we have

$$\frac{ \Vert \int_{X} f(x,\cdot) \,dx \Vert _{E^{p}}^{u}}{ \Vert \int_{X} g(x,\cdot) \,dx \Vert _{F^{q}}^{u-1}}\leq \frac{ (\int_{X} \Vert f(x,\cdot) \Vert _{E^{p}} \,dx )^{u}}{N^{1-u} (\int_{X} \Vert g(x,\cdot) \Vert _{F^{q}} \,dx )^{u-1}}, $$

and the statement follows by using reverse Hölder’s inequality.

Let $u<0$, *E* be 1-concave with constant of concavity equal to *M*, and let *F* be just a Banach function space. Then

$$\frac{ \Vert \int_{X} f(x,\cdot) \,dx \Vert _{E^{p}}^{u}}{ \Vert \int_{X} g(x,\cdot) \,dx \Vert _{F^{q}}^{u-1}}\leq \frac{M^{-u} (\int_{X} \Vert f(x,\cdot) \Vert _{E^{p}} \,dx )^{u}}{ (\int_{X} \Vert g(x,\cdot) \Vert _{F^{q}} \,dx )^{u-1}}, $$

and as before we use reverse Hölder’s inequality to complete the proof. □

### Remark 5.2

Inequality (5.1) can be rewritten as follows:

$$\frac{ \Vert (\int_{X} f(x,\cdot) \,dx )^{p} \Vert _{E}^{\frac{u}{p} }}{ \Vert (\int_{X} g(x,\cdot) \,dx )^{q} \Vert _{F}^{\frac{u-1}{q}}} \geq C \int_{X}\frac{ \Vert f^{p}(x,\cdot) \Vert _{E}^{\frac{u}{p}}}{ \Vert g^{q}(x,\cdot) \Vert _{F} ^{\frac{u-1}{q}}} \,dx. $$

Consider the case $E=F=L^{1}$. Then $M=N=1$, and we get the result which appears in Theorem 3.1 from [14].

### Remark 5.3

If we take *f* and *g* to be a step function of the type $f(x,y)=f_{1}(y)$ when $x\in[0,1), \ldots$ , $f(x,y)=f_{i}(y)$ when $x\in [i-1,i)$ for $i=2,3,\ldots,n$ and if $0< u<1$, if *E* is 1-concave with constant of concavity equal to *M*, if *F* is 1-concave with constant of concavity equal to *N*, then the inequality

5.2 $$ \frac{ \Vert \sum_{i=1}^{n}f_{i} \Vert _{E}^{u}}{ \Vert \sum_{i=1}^{n}g_{i} \Vert _{F}^{u-1}}\geq C\sum_{i=1}^{n} \frac{ \Vert f_{i} \Vert _{E}^{u}}{ \Vert g_{i} \Vert _{F}^{u-1}} $$

holds with $C=M^{-u}N^{1-u}$.

Next we state a kind of reverse version of Theorem 5.1, reversed in the same way as Popoviciu’s and Bellman’s inequalities may be regarded as reversed versions of Hölder’s and Minkowski’s inequalities, respectively.

### Theorem 5.4

*Let* $f_{0}(x)> \Vert f(x,\cdot) \Vert _{E^{p}}$ *for all* $x\in X$, *let* $g(x,z)$ *be a positive measurable function on* $X\times Z$ *and assume that* $g_{0}(x)$ *is a function on* *X* *such that* $g_{0}(x)> \Vert g(x,\cdot) \Vert _{F^{q}}$ *for all* $x\in X$, *where* *E* *is a Banach function space on* *Y* *for all* $x\in X$ *with the Fatou property and* *F* *is a Banach function space on* *Z* *for all* $x\in X$ *with the Fatou property*.

*If* $0< u< 1 $, $p\geq1 $ *or* $p<0$ *and* $q\geq1 $ *or* $q<0$, *then the following continuous reverse type version of Beckenbach*-*Dresher’s inequality holds*:

5.3 $$ \begin{aligned}[b] &\frac{ \{ [\int_{X}f_{0}(x) \,dx ]^{p}- \Vert [\int_{X} f(x,\cdot) \,dx ]^{p} \Vert _{E} \}^{\frac{u}{p}}}{ \{ [\int_{X}g_{0}(x) \,dx ]^{q}- \Vert [\int_{X} g(x,\cdot) \,dx ]^{q} \Vert _{F} \}^{\frac{u-1}{q}}} \\ &\quad \geq \int_{X}\frac{ [f^{p}_{0}(x)- \Vert f^{p}(x,\cdot ) \Vert _{E} ]^{\frac{u}{p}}}{ [g_{0}^{q}(x)- \Vert g^{q}(x,\cdot) \Vert _{F} ]^{\frac{u-1}{q}}} \,dx. \end{aligned} $$

*If* $u\geq1$, $0< p\leq1$ *and* $q\geq1 $ *or* $q<0$, *then reverse inequality* (5.3) *holds*.

*If* $u< 0$, $0< q\leq1$ *and* $p\geq1 $ *or* $p<0$, *then reverse inequality* (5.3) *holds*.

*In the cases when* $p<1$, *an additional condition on the function space* *E* *is that it should be concave with constant of concavity* 1; *in the cases when* $q<1$, *an additional condition on the function space* *F* *is that it should be concave with constant of concavity* 1.

### Proof

In the proof we use Theorem 4.1 and then Hölder’s inequality. Denote

$$\begin{aligned}& A= \biggl[ \int_{X}f_{0}(x) \,dx \biggr]^{p}- \biggl\Vert \biggl[ \int_{X} f(x,\cdot) \,dx \biggr]^{p} \biggr\Vert _{E}, \\& A_{1}(x)= \bigl[f^{p}_{0}(x)- \bigl\Vert f^{p}(x,\cdot) \bigr\Vert _{E} \bigr]^{\frac{1}{p}}, \\& B= \biggl[ \int_{X}g_{0}(x) \,dx \biggr]^{q}- \biggl\Vert \biggl[ \int_{X} g(x,\cdot) \,dx \biggr]^{q} \biggr\Vert _{F} \end{aligned}$$

and

$$B_{1}(x)= \bigl[g^{q}_{0}(x)- \bigl\Vert ^{q}(x,\cdot) \bigr\Vert _{E} \bigr]^{\frac{1}{q}}. $$

Consider the inequalities

$$\begin{aligned}& (1)\quad A\geq \biggl[ \int_{X} A_{1}(x) \,dx \biggr]^{p}, \\& (2) \quad A^{\frac{u}{p}}\geq \biggl[ \int_{X} A_{1}(x) \,dx \biggr]^{u}, \\& (3) \quad B\geq \biggl[ \int_{X} B_{1}(x) \,dx \biggr]^{q}, \\& (4) \quad B^{\frac{1-u}{p}}\geq \biggl[ \int_{X} B_{1}(x) \,dx \biggr]^{1-u}. \end{aligned}$$

Let $0< u\leq1$. If $p\geq1$, $q \geq1$, then our version (4.1) of Bellman’s inequality implies inequalities (1) and (3), and thus, since $\frac {u}{p}>0$ and $\frac{1-u}{q}\geq0$, inequalities (2) and (4) hold. Hence, by Hölder’s inequality

$$A^{\frac{u}{p}}B^{\frac{1-u}{q}}\geq \biggl[ \int_{X} A_{1}(x) \,dx \biggr]^{u} \biggl[ \int_{X} B_{1}(x) \,dx \biggr]^{1-u}\geq \int_{X} A_{1}(x)^{u}B_{1}(x)^{1-u} \,dx $$

and according to the definition of *A*, *B*, $A_{1}(x)$ and $B_{1}(x)$, inequality (5.3) is proved. If $p<0$, $q \geq1$, Bellman’s inequality gives (1) in the reverse direction, and since $\frac{u}{p}<0$, inequality (2) holds in this case, too. Since inequality (4) holds, we use Hölder’s inequality as above. The cases $p<0$, $q<0$ and $p\geq1$, $q<0$ can be done analogously.

Let now $u\geq1$. If $0< p\leq1$, then inequality (1) holds in the reverse direction and, therefore, since $\frac{u}{p}>0$, also (2) holds in the reverse direction. If $q\geq1$, then inequality (3) holds, and since $\frac{1-u}{q}\leq 0$ in this case, we conclude that (4) holds in the reverse direction. In the case $q<0$, inequality (3) holds in the reverse direction, but since $\frac{1-u}{q}\geq0$, inequality (4) holds in the reverse direction also in this case. The second statement thus follows by using reverse Hölder’s inequality and arguing as in the proof of the first case.

Finally, let $u<0$. If $p\geq1$, then again by Theorem 4.1 we have that inequality (1) holds, and since $\frac {u}{p}<0$ in this case, we find that inequality (2) holds in the reverse direction. Symmetrically, if $p<0$, inequality (1) holds in the reverse direction, but since $\frac{u}{p}>0$ in this case, still inequality (2) holds in the reverse direction.

If $0< q\leq1$, then inequality (3) yields in the reverse direction, and because $\frac{1-u}{q}\geq0$ in this case, also (4) holds in the reverse direction. The third case is thus proved by just using reverse Hölder’s inequality and arguing as in the first two cases. The proof is complete. □

## Concluding results

### Popoviciu type result in the case of infinite interpolation families

The result of this subsection was announced in [13] but here we give all the details.

Let D be a suitable simply connected domain in the complex plane with boundary Γ and $B(\gamma)\in\Gamma$ be an interpolation family on Γ in the sense of [15]. Let for simplicity $\Gamma=\{ \vert z \vert =1\}$ and $D=\{ \vert z \vert <1\}$. When we speak about interpolation in the families of Banach spaces (complex or real), we are in the situation when the actual family of Banach spaces is indexed by the points of the unit circle for simplicity $\Gamma=\{ \vert z \vert =1\}$ in the complex plane, while the interpolation spaces are labeled by the points of the unit disk $D=\{ \vert z \vert <1\}$. The authors of [15] construct, for each $z_{0} \in D$, a new space $B [z_{0}]$, which consists of the values $f(z_{0})$ at $z_{0}$ of certain analytic vector-valued functions $f(z)$ on *D* whose boundary values $f(\gamma) \in B(\gamma)$ for a.e. $\gamma\in\Gamma$, and $\Vert f \Vert _{F(\gamma)}=\operatorname{ess}\sup_{\gamma} \Vert f(\gamma) \Vert _{B(\gamma)}<\infty$. The space $B [z_{0}]$ has an interpolation property, i.e., if a linear operator T is bounded on each $B(\gamma)$ with norm $M(\gamma)$ and also bounded on a certain space *U* containing each $B(\gamma)$, then T is also bounded on $B [z_{0}]$ with norm $M(z_{0})$, which can be estimated in terms of the function $M(\gamma)$. A variant of the construction was suggested independently in [16].

Proposition 2.4 from [15] says that, for each $f\in{F(\gamma)}$ and each $z_{0}\in D$, the inequality

6.1 $$ \bigl\Vert f(z_{0}) \bigr\Vert _{B [z_{0}]}\leq \exp \biggl( \int_{\Gamma}\log \bigl\Vert f(\gamma) \bigr\Vert _{B(\gamma)} \,dP_{z_{0}}(\gamma) \biggr) $$

holds, where $P_{z_{0}}(\gamma)$ is the Poisson kernel. This can be regarded as an infinite variant of the inequality (log convexity inequality) in the notion of the exact interpolation method of type *θ* (here $z_{0}$) in the case when the families consist of just two spaces (the case of Banach couples).

In such terms we are now ready to formulate the following general Popoviciu type inequality.

#### Theorem 6.1

*Let* $B(\gamma)$, $\gamma\in\Gamma$ *be an interpolation family on* Γ, *let* $f\in{F(\gamma)}$ *and* $z_{0}\in D$ *and* $B [z_{0}]$ *be the complex interpolation space*. *If* $f_{0}> \Vert f \Vert _{F(\gamma)}=\operatorname{ess}\sup_{\gamma} \Vert f(\gamma) \Vert _{B(\gamma)}>0$, *then*

$$f_{0}- \bigl\Vert f(z_{0}) \bigr\Vert _{B [z_{0}]} \geq \exp \biggl( \int _{\Gamma}\log\bigl[f_{0}- \bigl\Vert f(\gamma) \bigr\Vert _{B(\gamma)} \bigr] \,dP_{z_{0}}(\gamma) \biggr). $$

#### Proof

Having in mind inequality (6.1), we find that

$$\begin{gathered} \bigl\Vert f(z_{0}) \bigr\Vert _{B [z_{0}]}+ \exp \biggl( \int_{\Gamma}\log \bigl[f_{0}- \bigl\Vert f(\gamma) \bigr\Vert _{B(\gamma)} \bigr] \,dP_{z_{0}}(\gamma ) \biggr) \\ \quad \leq\exp \biggl( \int_{\Gamma}\log \bigl\Vert f(\gamma) \bigr\Vert _{B(\gamma)} \,dP_{z_{0}}(\gamma) \biggr)+\exp \biggl( \int_{\Gamma}\log \bigl[f_{0}- \bigl\Vert f(\gamma) \bigr\Vert _{B(\gamma)} \bigr] \,dP_{z_{0}}(\gamma ) \biggr) \\ \quad \leq\exp \biggl( \int_{\Gamma}\log f_{0} \,dP_{z_{0}}(\gamma) \biggr)=f_{0}, \end{gathered} $$

which gives the stated inequality. Here we used inequality from Lemma 2.3 in a crucial way. □

### Connection to Milne’s inequality

Denote $X=\sqrt{(c_{1}^{2}- \Vert f^{2} \Vert ) (c_{2}^{2}- \Vert g^{2} \Vert )}$. Then if in the particular finite case (3.3) put $p=q=2$, we find that

6.2 $$ X\leq c_{1}c_{2}-\sqrt{ \bigl\Vert f^{2} \bigr\Vert )}\sqrt{ \bigl\Vert g^{2} \bigr\Vert }. $$

From this inequality and from Hölder’s inequality, we obtain the inequality

6.3 $$ X\leq c_{1}c_{2}- \Vert fg \Vert . $$

Consider Milne’s inequality [17]

$$\sum_{i=1}^{2} \bigl(a_{i}^{2}+b_{i}^{2} \bigr) \sum_{i=1}^{2} \frac{a_{i}^{2}b_{i}^{2}}{a_{i}^{2}+b_{i}^{2}} \leq\sum_{i=1}^{2} a_{i}^{2} \sum_{i=1}^{2} b_{i}^{2} $$

and put

$$a_{1}=\bigl(c_{1}^{2}- \bigl\Vert f^{2} \bigr\Vert \bigr)^{1/2},\qquad b_{1}= \bigl(c_{2}^{2}- \bigl\Vert g^{2} \bigr\Vert \bigr)^{1/2}, \qquad a_{2}=\bigl( \bigl\Vert f^{2} \bigr\Vert \bigr)^{1/2},\qquad b_{2}=\bigl( \bigl\Vert g^{2} \bigr\Vert \bigr)^{1/2}. $$

Since $X=\sqrt{a_{1}b_{1}}$, we can estimate it and get

6.4 $$ \bigl(c_{1}^{2}- \bigl\Vert f^{2} \bigr\Vert \bigr) \bigl(c_{2}^{2}- \bigl\Vert g^{2} \bigr\Vert \bigr)\leq \biggl[\frac {c_{1}^{2}c_{2}^{2}}{c_{1}^{2}+c_{2}^{2}}- \frac{ \Vert f^{2} \Vert \Vert g^{2} \Vert }{ \Vert f^{2} \Vert + \Vert g^{2} \Vert } \biggr] \bigl[c_{1}^{2} +c_{2}^{2}- \bigl\Vert f^{2} \bigr\Vert - \bigl\Vert g^{2} \bigr\Vert \bigr]. $$

It is easy to see that this inequality is stronger than (6.2).

In fact, we can use Hölder’s inequality here too and get

6.5 $$ \bigl(c_{1}^{2}- \bigl\Vert f^{2} \bigr\Vert \bigr) \bigl(c_{2}^{2}- \bigl\Vert g^{2} \bigr\Vert \bigr)\leq \biggl[\frac {c_{1}^{2}c_{2}^{2}}{c_{1}^{2}+c_{2}^{2}}- \frac{ \Vert fg \Vert ^{2}}{ \Vert f^{2} \Vert + \Vert g^{2} \Vert } \biggr] \bigl[c_{1}^{2} +c_{2}^{2}- \bigl\Vert f^{2} \bigr\Vert - \bigl\Vert g^{2} \bigr\Vert \bigr]. $$
